# Invasive Tracheal and Cranial Mediastinal Aspergillosis in a Young Otherwise Healthy Cat

**DOI:** 10.1111/jvim.70204

**Published:** 2025-08-13

**Authors:** Liliana M. Mutascio, J. Clifton Crooks

**Affiliations:** ^1^ VetMED Emergency and Specialty Veterinary Hospital Phoenix Arizona USA

**Keywords:** *Aspergillus Lentulus*, cranial mediastinal mass, fungal granuloma

## Abstract

A 3.5‐year‐old castrated male domestic medium hair cat was evaluated for dry cough and labored breathing. A cranial mediastinal mass was seen on thoracic radiographs. On computed tomography, the mass displaced the cranial vena cava and dorsally displaced and compressed the intrathoracic trachea. The patient was taken to surgery for attempted mass removal. Intraoperatively, the mass was adhered to the cranial vena cava, aortic arch, left subclavian artery, and had partially engulfed the brachiocephalic trunk. The cat was euthanized and on necropsy the mass was found to invade the lumen of the vena cava and the tracheal lumen. An *Aspergillus lentulus* fungal granuloma was diagnosed histologically and by fungal culture and PCR. We highlight the difficulty in determining the extent of invasion with invasive aspergillosis and provide evidence that invasive aspergillosis can occur in otherwise healthy, young cats with no concurrent immunosuppressive treatments or comorbidities.

**Abbreviations**
BLAST: Basic Local Alignment Search Tool
bp: base pairs
cm: centimeter
CT: computed tomography
DIA: disseminated invasive aspergillosus
mg/kg/h: milligram per kilogram per hour
NCBI: National Center for Biotechnology Information
PT/PTT: prothrombin time and partial thromboplastin time

## 1 Case Report

A 3.5‐year‐old, 5.3 kg castrated male domestic medium hair cat was presented for evaluation of a 10‐day history of dry cough, labored breathing, lethargy, hyporexia, and a previously identified cranial mediastinal mass. There was no history of travel outside of Arizona. A CBC and serum biochemistry panel performed one week before evaluation was normal, and feline immunodeficiency virus antibody and feline leukemia virus point‐of‐care ELISA assays (IDEXX, USA) were negative. Radiographs of the cervical region, thorax, and abdomen performed by the referring veterinarian identified a large mass in the ventral portion of the cranial mediastinum, causing dorsal deviation and severe focal narrowing of the middle one‐third portion of the thoracic trachea. On physical examination, the cat's respiratory rate was increased (50 breaths/min) and respiratory effort was intermittently increased. Body condition score was 5/9; temperature was 97.3°F (axillary), and pulse was 220 beats/min. Bronchopulmonary sounds were harsh with referred wheezes. Chest compressibility was subjectively decreased cranially. Prothrombin time and partial thromboplastin time (PT/PTT) were within normal limits.

Computed tomography (CT) imaging was performed the next day using a GE Brightspeed 16‐slice CT scanner. A pre‐contrast CT series of the thorax and abdomen was performed, and data volume acquisitions were reconstructed in standard and detail with algorithms at 2.5 mm slice thickness. After acquisition of the initial series, 240 mg/mL iohexol was administered IV at a dose of 600 mg/kg, and arterial phase and venous phase post‐contrast scans of the thorax and a 1‐min 20‐s delayed scan of the thorax and abdomen (reconstructed in detail and soft tissue algorithms at 1.25 mm slice thickness) were made. Image assessment was performed by a board‐certified veterinary radiologist.

The CT study showed an ovoid, diffusely soft tissue‐attenuating (Hounsfield units: approximately 40–60), partially peripherally (rim) contrast‐enhancing but mostly noncontrast enhancing mass filling most of the cranial mediastinum, measuring approximately 50 mm in length and 40 mm in width and height. The caudal‐most portion of the mass was located between the right and left pulmonary veins (immediately dorsal to the point of communication and insertion into the left atrium), just ventral to the carina, and dorsoaxial to the aortic arch (with the cranial aspect of the descending aorta coursing along the left lateral margin of the mass). This mass extended cranially to the level of the second intercostal space, resulting in marked ventral and slight rightward displacement of the cranial vena cava; moderate left ventrolateral displacement of the right brachiocephalic artery, right subclavian artery, and right and left common carotid arteries; and, moderate left lateral displacement of the left subclavian artery. No persistent contrast‐filling defects or abnormal contrast enhancement suggestive of intraluminal invasion by the mass or thrombosis were present in the intrathoracic vasculature. There was also moderate to marked dorsal displacement of the intrathoracic trachea, with the dorsal aspect of the mass encircling and appearing to severely compress the trachea and narrow it for an approximately 20 mm segment of the mid‐thoracic trachea, with worse luminal narrowing on the left. Definitive evidence of extension of the mass into the tracheal lumen was not identified. The remaining mediastinal structures and space, aside from left lateral deviation of the cranial two‐thirds of the intrathoracic esophagus, were unremarkable. Differential diagnosis for this mass included neoplasia (e.g., lymphoma, thymoma, carcinoma), granuloma, reactive lymphadenopathy, or lymphadenitis.

Two irregularly shaped focal soft tissue attenuating regions were observed in the left cranial lung lobe, one in the caudal subsegment (measuring 15 mm length × 13 mm width × 10 mm height) and the other in the cranial subsegment (measuring 10 mm length × 5 mm width × 12.5 mm height). Focal atelectasis secondary to mass effect was suspected, but fibrosis, granulomatous lesions, multifocal pneumonia, or, less likely, pulmonary metastatic lesions also were considered. No other abnormalities of the thorax or abdomen were present.

Immediately after the CT examination, ultrasound‐guided fine needle aspiration of the cranial mediastinal mass was performed. The mass was moderately heterogeneous (mottled) on ultrasound evaluation before aspiration. No color flow was noted on color or power Doppler evaluation of the mass. The cat recovered uneventfully and was discharged the same day. Cytology findings included a moderate amount of amorphous debris and rare polygonal cells with abundant basophilic cytoplasm, most consistent with mesothelial cells. Findings were interpreted as necrosis and were inconclusive for neoplasia versus other causes.

The cat presented to the emergency department 5 days after the CT scan for acute respiratory distress and was placed in an oxygen kennel (Snyder Mfg. Co Intensive Care Unit, Snyder Manufacturing Company, Centennial, CO) at 40% oxygen. Butorphanol (0.2 mg/kg IV) was administered q4h as needed for sedation. Thoracic radiographs (3‐view) showed no evidence of aspiration pneumonia. Because of worsening respiratory signs, urgent surgical treatment was pursued for mass removal. The cat was premedicated with fentanyl (5 μg/kg IV) and midazolam (0.2 mg/kg IV). After induction with propofol (6 mg/kg IV), general anesthesia was maintained using isoflurane in 100% oxygen. Continuous rate infusions of fentanyl (10 μg/kg/h IV) and ketamine (0.6 mg/kg/h) also were administered. A standard median sternotomy was performed for attempted cranial mediastinal mass removal. No free fluid was found in the thoracic cavity. The mass extended from the cranial‐most aspect of the thorax to just caudal to the aortic arch. The trachea was not visible or palpable dorsal to the mass. The left cranial lung lobe, cranial vena cava, left subclavian artery, brachiocephalic trunk, and aortic arch were adhered to the mass. The brachiocephalic trunk was partially embedded within the mass. The left subclavian artery was successfully separated from the mass using blunt dissection. During dissection of the brachiocephalic trunk, the vessel wall tore and suture repair was unsuccessful. Given the grave prognosis, the owner elected for euthanasia, and necropsy was performed.

At necropsy, the cranial mediastinal mass was found to be segmentally effacing and infiltrating the distal tracheal wall. The distal tracheal lumen was approximately 75% narrowed and more caudally was flattened to an elliptical shape. The brachiocephalic trunk was segmentally compressed and surrounded by the mass. The cranial vena cava contained a 0.2 cm diameter nodule that extended from the mass into the vessel lumen. Invasion of the tracheal wall by the mass was confirmed histologically. Fibrous connective tissue, inflammatory cells, and cellular debris from the mass were found to be infiltrating into all layers of the tracheal wall. Fungal plaques were seen protruding into the tracheal lumen, and fungal hyphae were found migrating into the perichondrium and tracheal cartilage. Invasion by the mass into multiple additional adjacent structures, including tracheobronchial and subjacent lymph nodes, nerves, cranial vena cava adventitia, other blood vessels, and pulmonary tissue, also was histologically noted. Histopathologic evaluation of lung tissue confirmed atelectasis with no hyphal invasion.

The mass was diagnosed as a large fibrosing fungal granuloma causing severe, chronic, locally extensive, eosinophilic, and granulomatous tracheitis and mediastinitis with many intralesional fungal organisms. Two fungal organisms of different morphology were identified histologically, one hyphal and one pseudohyphal. On fungal culture, the hyphal fungus *Aspergillus lentulus* was identified; no pseudohyphal fungus was identified on culture. Panfungal PCR was performed and confirmed infection with *A. lentulus*. According to the PCR report, panfungal PCR targeting the internal transcribed spacer (ITS) region and agarose gel electrophoresis yielded 1 band of DNA of approximately 300 base pairs (bp). The long subunit region (LSU) also was amplified, and agarose gel electrophoresis yielded 3 bands of DNA of approximately 100 bp, 400 bp, and 1000 bp. The DNA was purified from the gel and sequenced. The resulting 343 bp and 918 bp contig sequence was analyzed using the National Center for Biotechnology Information (NCBI) Basic Local Alignment Search Tool (BLAST) database. The sequence matched *A. lentulus* with 99.70% and 99.89% identity. The LSU region also matched *Aspergillus oerlinghausenensis* and *Aspergillus fumisynnematus* with 100% identity.

## 2 Discussion

In this case report, we describe focal invasive tracheal and cranial mediastinal disease in a young cat with no comorbidities. Despite a lack of concurrent immunosuppressive disease or immunosuppressive treatment in this cat, a severe form of aspergillosis developed with life‐threatening invasion of the trachea and major thoracic vasculature. The lack of obvious invasion into these structures on preoperative radiographs and CT angiography highlights an uncommon but severe diagnosis that ultimately should be included in the differential diagnosis for patients of similar signalment presented for evaluation of a cranial mediastinal mass.

**FIGURE 1:**
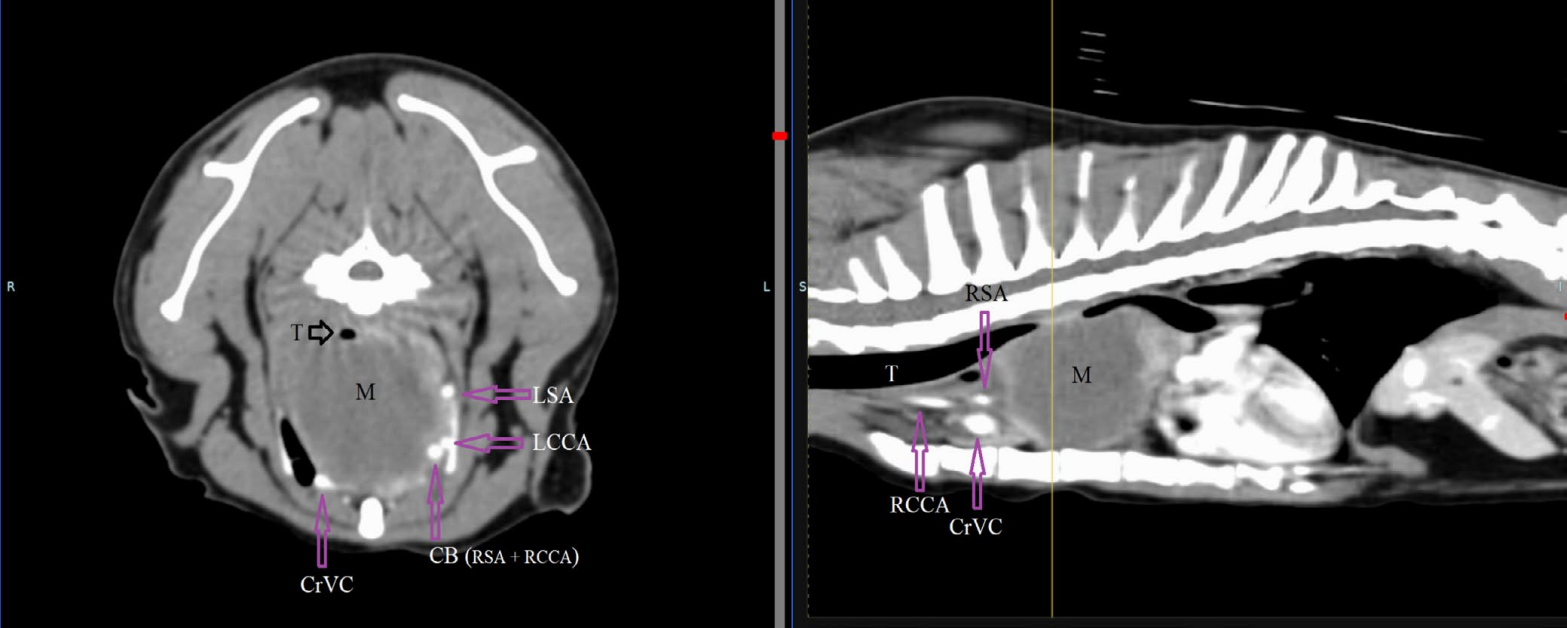
CT: Left parasagittal of midline image and transverse image at point of probable invasion into the trachea (T).

**FIGURE 2:**
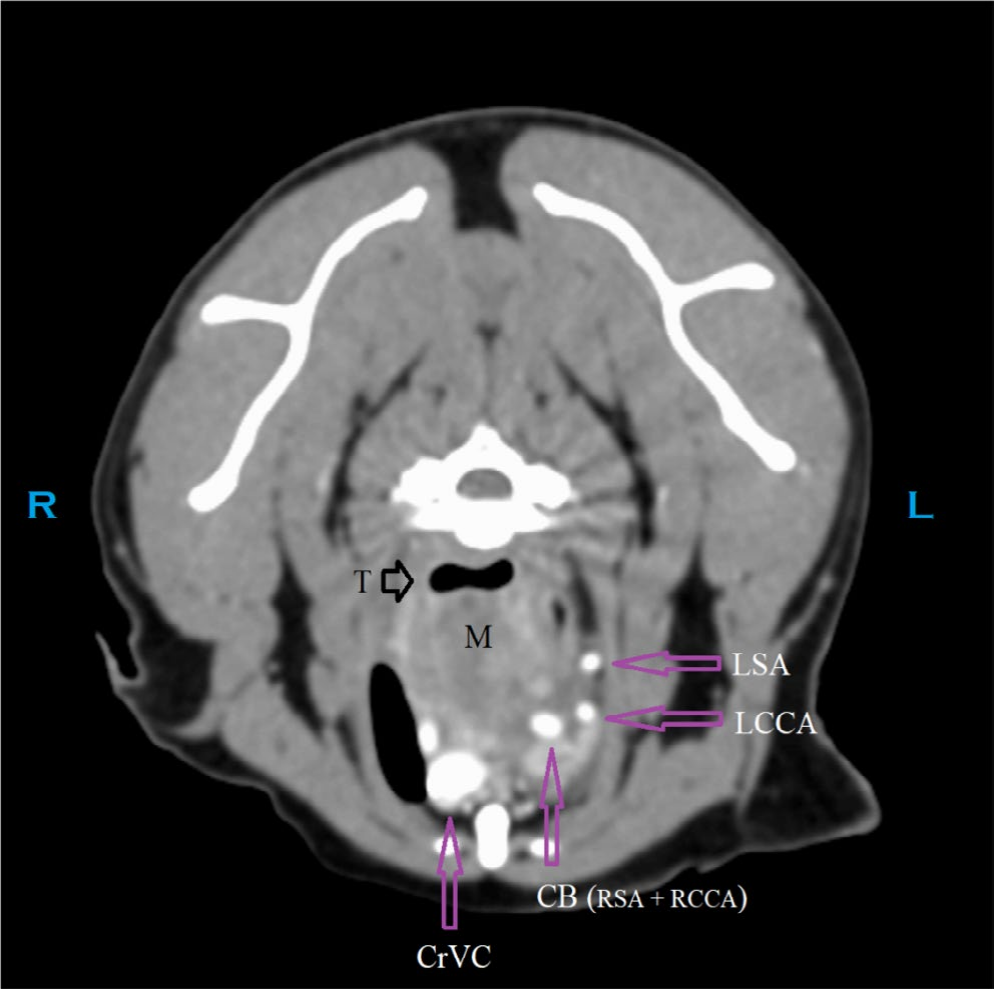
CT: Transverse image cranial to the point of probable invasion into the trachea (T).

**FIGURE 3:**
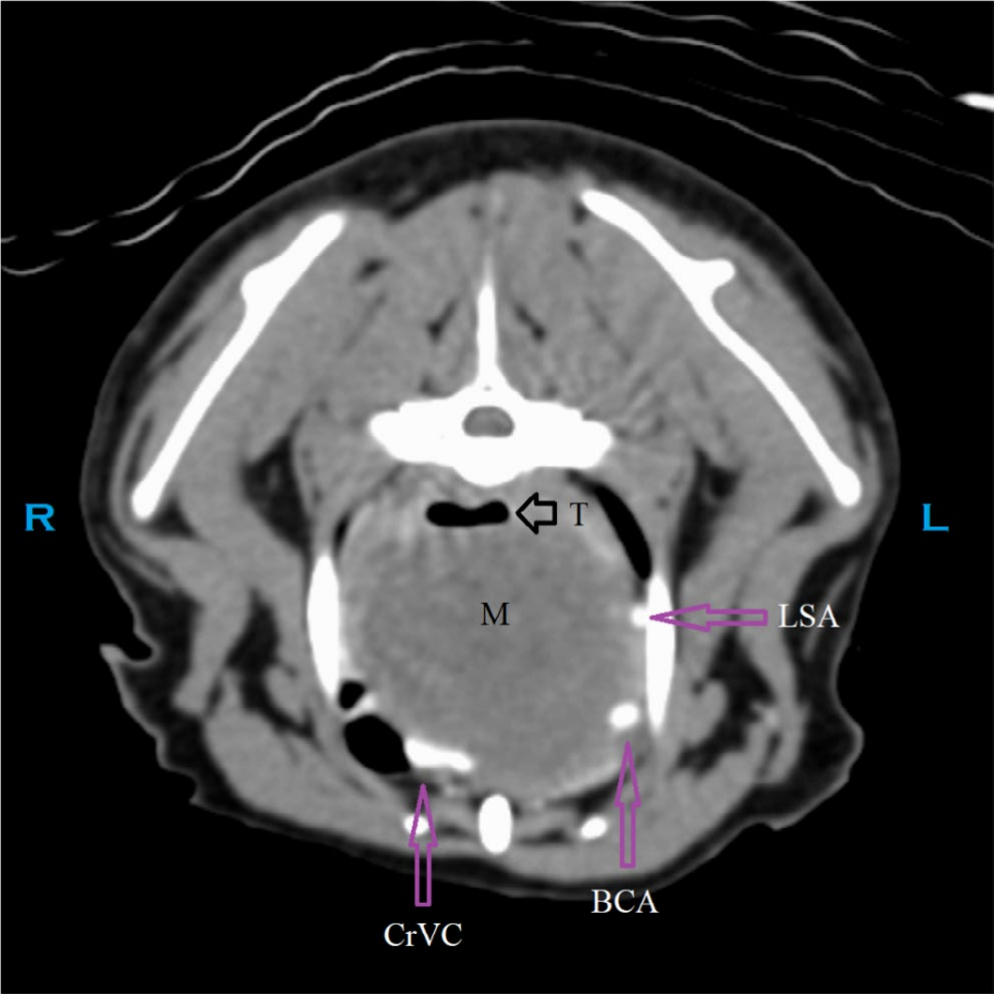
CT: Transverse image caudal to the point of probable invasion into the trachea (T). BCA, brachiocephalic artery; CB, common branch for the right subclavian artery and right common carotid artery [1]; CrVC, cranial vena cava; L, left; LCCA, left common carotid artery; LSA, left subclavian artery; M, mass; R, right; RCCA, right common carotid artery; RSA, right subclavian artery; T, trachea.

Although not noted surgically or identified at necropsy, the point of invasion by the cranial mediastinal mass into the airway is retrospectively suspected to have been at the narrowed segment of the mid‐thoracic trachea, at or near the region identified in Figure 1. The poor delineation of this invasion on CT may be the result of minimal or partial contrast enhancement of the mass, lack of delineation of the margins of the trachea versus the surrounding mass, possible concurrent extra‐luminal compression on the trachea by the mass resulting in additional narrowing of the lumen, the presence of a progressive decrease in luminal diameter instead of an overt focal space‐occupying intraluminal structure, or some combination of these factors (Figures 2 and 3).

*Aspergillus* fungi are ubiquitous in the environment and can cause several types of disease in dogs, cats, and humans, including localized sinonasal infection, disseminated invasive bronchopulmonary or systemic disease, and keratomycosis [2, 3, 4]. *Aspergillus* infections are more common in dogs than in cats, and sinonasal infection is the most commonly encountered type of disease [2, 5, 6]. Although sinonasal infection typically is non‐invasive in both species, the overall prognosis for resolution in cats with localized sinonasal infection is worse than in dogs, and the prognosis for cats with invasive sino‐orbital infection is poor [7, 8]. Aggressive surgical debridement of invasive sino‐orbital disease in cats has been reported in a small number of cases, but outcomes were not different with or without debridement [7, 8].

Disseminated invasive aspergillosis (DIA) also carries a poor prognosis for both dogs and cats. The disease spreads by a hematogenous route after inhalation of *Aspergillus* conidia and can affect any organ. Dogs with disseminated invasive disease have been reported to have a cranial mediastinal mass on thoracic radiographs, and rare body cavity infections in DIA have been attributed to penetrating injuries [9, 10]. Although the cat in the present report had a cranial mediastinal mass, no evidence of dissemination was found on necropsy. The cat did not have a history of penetrating intrathoracic wounds or other injury. The most likely source of infection in the cat was tracheal aspergillosis that extended through the tracheal wall and into the cranial mediastinum. This type of invasion across tissue planes also is seen with sino‐orbital aspergillosis in cats. Tracheal granulomas caused by *Aspergillus* species have been previously reported in birds [11].

Treatment options in cats with invasive aspergillosis or DIA have not been evaluated, but rare reports of recovery have been described where the invasive disease was more localized and the affected tissue could be surgically resected [12, 13, 14, 15, 16]. In the present case, surgical resection would not have been possible given the invasion of fungal plaques into the trachea and cranial vena cava. Strong evidence of invasion was not present on preoperative imaging for this cat, and the decision to perform surgery was made to attempt mass removal and alleviate the worsening clinical signs. If tracheal or vena caval invasion or both had been documented on CT before surgery, surgical treatment likely would not have been recommended.

Our case report emphasizes the difficulty in determining the extent of invasiveness in a cat with aspergillosis, even with preoperative CT. It also highlights that invasive aspergillosis should be included in the differential diagnosis for young cats with a cranial mediastinal mass, regardless of the absence of concurrent immunosuppression or other illnesses. The outcome in the cat of our report supports the previously documented poor prognosis in cats with invasive aspergillosis, especially in cases where surgical resection is not possible. An important feature of our case was the lack of visible invasion on preoperative imaging. This aggressive presentation of aspergillosis warrants consideration for any young cat presenting with a cranial mediastinal mass, and although rare, clinicians should keep this diagnosis and its associated poor prognosis in mind when approaching cats with similar signalment.

## Disclosure

Authors declare no off‐label use of antimicrobials.

## Ethics Statement

Informed consent (verbal and written) was obtained from the owner of the animal described in this work for all procedures undertaken. Authors declare human ethics approval was not needed.

## Conflicts of Interest

The authors declare no conflicts of interest.
